# Safety and Efficacy of 188-Rhenium-Labeled Antibody to Melanin in Patients with Metastatic Melanoma

**DOI:** 10.1155/2013/828329

**Published:** 2013-01-10

**Authors:** M. Klein, M. Lotem, T. Peretz, S. T. Zwas, S. Mizrachi, Y. Liberman, R. Chisin, J. Schachter, I. G. Ron, G. Iosilevsky, J. A. Kennedy, E. Revskaya, A. W. de Kater, E. Banaga, V. Klutzaritz, N. Friedmann, E. Galun, G. L. DeNardo, S. J. DeNardo, A. Casadevall, E. Dadachova, G. B. Thornton

**Affiliations:** ^1^Hadassah Medical Center, Hebrew University, Kiryat Hadassah, 91120 Jerusalem, Israel; ^2^Chaim Sheba Medical Center, Tel Hashomer, 52621 Ramat Gan, Israel; ^3^Sackler Faculty of Medicine, Tel Aviv Medical Center, 69978 Tel Aviv, Israel; ^4^Rambam Health Care Campus, 31096 Haifa, Israel; ^5^Albert Einstein College of Medicine, 1300 Morris Park Avenue, Bronx, NY 10461, USA; ^6^Pain Therapeutics, Inc., Austin, TX 78731, USA; ^7^Davis Medical Center, University of California, Sacramento, CA 95817, USA

## Abstract

There is a need for effective “broad spectrum” therapies for metastatic melanoma which would be suitable for all patients. The objectives of Phase Ia/Ib studies were to evaluate the safety, pharmacokinetics, dosimetry, and antitumor activity of ^188^Re-6D2, a 188-Rhenium-labeled antibody to melanin. Stage IIIC/IV metastatic melanoma (MM) patients who failed standard therapies were enrolled in both studies. In Phase Ia, 10 mCi ^188^Re-6D2 were given while unlabeled antibody preload was escalated. In Phase Ib, the dose of ^188^Re-6D2 was escalated to 54 mCi. SPECT/CT revealed ^188^Re-6D2 uptake in melanoma metastases. The mean effective half-life of ^188^Re-6D2 was 12.4 h. Transient HAMA was observed in 9 patients. Six patients met the RECIST criteria for stable disease at 6 weeks. Two patients had durable disease stabilization for 14 weeks and one for 22 weeks. Median overall survival was 13 months with no dose-limiting toxicities. The data demonstrate that ^188^Re-6D2 was well tolerated, localized in melanoma metastases, and had antitumor activity, thus warranting its further investigation in patients with metastatic melanoma.

## 1. Introduction

The incidence of melanoma is increasing worldwide, with a concomitant rise in mortality from metastatic disease. Patients who progress to stage IV metastatic melanoma (MM) have a median survival of less than 1 year [1]. In the United States, about 9,180 people will die from melanoma in 2012 (American Cancer Society, 2012). Until recently, treatment options for patients with stage IV disease were limited and offered marginal, if any, improvement in overall survival. This situation changed with the newly approved by FDA ipilimumab (anti-CTLA4 monoclonal antibody), an immunomodulator which in a phase III trial was shown to improve overall survival [2]. In addition, vemurafenib that inhibits mutated B-RAF protein offers hope for 40–60% melanoma patients carrying this mutation [3, 4]. However, the responses to the latter have been relatively short lasting followed by recurrences.

In our search for alternative therapeutic options for MM we turned to radioimmunotherapy (RIT). RIT takes advantage of the specificity of the antigen-antibody interaction to deliver cytotoxic radiation to tumors [5, 6]. The clinical success of FDA-approved drugs such as ibritumomab tiuxetan (anti-CD20 monoclonal antibody (mAb) labeled with ^90^Yttrium) for treatment of primary, relapsed or refractory B-cell non-Hodgkin lymphoma demonstrates RIT potential as antineoplastic strategy. Unlike other therapies, RIT does not rely on specific genotypes, biochemical pathways, or the variability of an individual's immune response and thus can be offered to broad patient populations and is not a subject to multidrug resistance mechanisms which are already limiting the efficacy of vemurafenib.

Melanoma owes its name to melanin pigment with even “amelanotic” melanomas containing some melanin [7, 8]. Historically, melanin was not considered a target for RIT because of its intracellular location in melanosomes beyond the reach of melanin-specific mAbs. However, in rapidly growing melanoma tumors cell necrosis releases melanin into the extracellular space where it can be targeted for delivery of radiation by radiolabeled melanin-binding mAbs. Several mAbs to fungal melanin were generated in our laboratories [9]. We established the feasibility of targeting melanin in melanoma xenografts with melanin-binding mAb 6D2 IgM labeled with beta-emitting radionuclide ^188^Re (*E*_max⁡_ = 2.1 MeV, half-life 17.0 hrs) [10]. In spite of their fast clearance from the circulation which should result in favorable target to nontarget ratios, IgMs are often overlooked in radioimmunoimaging and RIT. Importantly, experiments with melanin-binding mAbs conducted in C57Bl6 black mice demonstrated that melanin in normal pigmented tissues such as retina of the eye, pigmented skin, or hair follicles is not accessible to the mAb by virtue of its intracellular location [10]. Preclinical development of ^188^Re-6D2 resulted in developing cGMP-compatible radiolabeling methodology while computer-simulated tumor dosimetry demonstrated that ^188^Re-6D2 could deliver tumoricidal doses to tumors within the wide range of melanin concentrations (up to 100 less melanin than in primary tumors) [11, 12]. Here we report the results of the recently completed consecutive Phase Ia and Ib trials of ^188^Re-6D2 mAb in patients with MM.

## 2. Materials and Methods

### 2.1. Patients Eligibility and Screening

Patients were eligible for enrollment if they had histologically or clinically confirmed stage III (unresectable) or stage IV metastatic melanoma. The studies were conducted in accordance with International Conference on Harmonization guidelines and Good Clinical Practice guidelines. The studies were registered at clinicaltrials.gov and were numbered NCT00399113 and NCT00734188. The study protocols were approved by the Ethics Committees at Hadassah Medical Center, Jerusalem and Sheba Medical Center, Tel Hashomer, Israel where the study was conducted. All patients signed a written informed consent prior to participating in the study. Men and women (not pregnant or lactating, and following acceptable methods of birth control) were to be 18 years or older and have a life expectancy of at least 3 months. At least 4 weeks had to have elapsed since prior chemotherapy or radiation therapy and at least 1 week since IL-2 therapy. Patients had to have failed response to at least one previous therapy; adequate organ and marrow function and a negative human anti-mouse antibody (HAMA) result; no cerebral metastases by MRI or CT; no ocular diseases that may have led to an impaired blood-retinal brain barrier and no prior parenteral exposure to murine proteins.

### 2.2. Antibody, Radioisotope, and Radiolabeling

MAb 6D2, a murine IgM generated against fungal melanin, was previously described in references [8–12]. The clinical lot of 6D2 and all reagents used for manufacturing were produced in compliance with cGMPs by Goodwin Biotechnology Inc. (Plantation, FL). ^188^Re was obtained from a ^188^W/^188^Re generator (Oak Ridge National Laboratory, Oak Ridge, TN, USA). The radiolabeling of 6D2 with ^188^Re was performed as in [11]. The specific activity of ^188^Re-6D2 in both Phase I studies was kept approximately at 1 mCi/mg to preserve 6D2 immunoreactivity towards melanin as was demonstrated in reference [11]. The highest level of activity achievable with ^188^W/^188^Re generators used in the study was 60 mCi/50 mg.

### 2.3. Phase I Study Objectives and Design

Both open-label Phase I studies had the following objectives: (1) characterization of pharmacokinetics and dosimetry to normal organs; (2) identification of the dose-limiting organs; (3) evaluation of the HAMA response; (4) determination of safety and tolerability; (5) evaluation of tumor localization and antitumor activity of ^188^Re-6D2. The first Phase I study (phase Ia) had an additional objective of determining the effect of preload with “cold” (unlabeled) mAb on the biodistribution and pharmacokinetics of ^188^Re-6D2 while the second phase I (phase Ib) study evaluated possible toxicities associated with increasing radioactive doses of ^188^Re-6D2. Phase Ia enrolled 13 patients into 4 cohorts with each cohort receiving 10 mCi/10 mg ^188^Re-6D2 preceded by either 0, 10, 20, or 50 mg of unlabeled 6D2 depending upon cohort. Seven patients in the Phase Ib were enrolled into 2 cohorts: 20–30 mCi and 40–60 mCi. Patients returned for posttreatment followup at 2 and 6 weeks after infusion (Phase Ia and Ib) and every 8 weeks thereafter until disease progression (Phase Ib).

### 2.4. Pharmacokinetics, Imaging, and Dosimetry for the Normal Organs

MAb pharmacokinetics was determined from blood samples taken at specified time intervals from predose to 48 h after ^188^Re-6D2 administration. Biodistribution of ^188^Re-6D2 was evaluated by whole body planar imaging. SPECT/CT scans were performed for the regions of interest after each planar imaging session as necessary. The percentages injected dose for the organs and whole body were calculated from regions of interest and fitted to an exponential kinetic model within the dosimetry code Organ Level Internal Dose Assessment (OLINDA) [13, 14].

### 2.5. Tumor Response Assessment

Tumor response (based upon evaluation of target, nontarget, and emergence of new lesions) was assessed utilizing RECIST version 1.0. Cutaneous melanoma lesions were measured and the longest diameter recorded in millimeters. Noncutaneous lesions were identified by radiologic assessment and measured. Tumors were recorded at baseline and tumor response was monitored at 2 and 6 weeks after infusion. In the Phase Ib, tumors continued to be monitored every 8 weeks until disease progression. In addition, whole body ^18^FDG PET/CT scans were performed at baseline and approximately 2 and 6 weeks after infusion to observe the metabolic activity of the tumors.

### 2.6. Safety

Safety was evaluated by adverse event monitoring, vital signs, physical examinations, ECGs, and clinical laboratory tests which included HAMA and thyroid function. Safety assessments were part of patients followup.

### 2.7. Statistical Analysis

This study was not powered for conclusions regarding efficacy. Data were summarized descriptively. Data from patients receiving any amount of ^188^Re-6D2 were included in safety summaries. A post hoc pooled analysis of overall survival was conducted among 17 subjects (12 from Phase Ia and 5 from Phase Ib). Overall survival, which was not a planned endpoint in either trial, was calculated as the time from ^188^Re-6D2 administration until death. Subjects lost to followup or alive at the time of data cutoff were censored at the date of last contact. Median survival was estimated using Kaplan-Meier methods.

## 3. Results

### 3.1. Patient Demographics and Cohort Assignment

Hadassah Medical Center and Sheba Medical Center enrolled 20 MM patients who met the study eligibility criteria with 13 stage IV patients enrolled into Phase Ia and 7 stage IIIC/IV patients—into Phase Ib.  Table 1 presents the patient demographic data. All patients had active metastatic disease at the time of accrual, and all had failed at least two previous treatments including Dacarbazine-containing regimens (13 patients) or ambulatory doses of IL-2 (4 patients).

In phase Ia all 13 patients received 10 mCi ^188^Re-6D2 and an infusion of unlabeled 6D2 depending upon the cohort entered. Three patients entered cohort 1 and did not receive an infusion of unlabeled 6D2, 4 patients (cohort 2) received 10 mg 6D2, 3 patients (cohort 3) received 20 mg, and 3 patients (cohort 4)—50 mg. Two patients in cohort I received a second 10 mCi dose of ^188^Re-6D2. In phase Ib, 4 patients (cohort 1) received 20–30 mCi ^188^Re-6D2 and 3 patients (cohort 2)—41–54 mCi ^188^Re-6D2.

### 3.2. Tumor Imaging and Absence of ^188^Re-6D2 Localization in Normal Melanized Tissues

**Figure 1:**
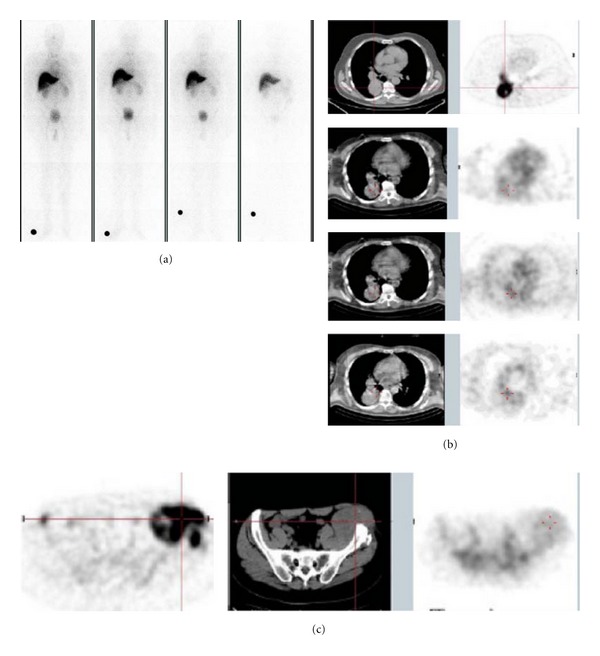
Imaging of patients with ^188^Re-6D2 mAb. (a) Whole-body scintigraphic image of a patient from Phase Ia showing absence of ^188^Re-6D2 uptake in normal melanized tissues: from left to right—0.4, 3, 5, and 20 hr images; (b) patient from Phase Ia study with mediastinal and lung metastases: top panel—^18^FDG PET/CT 10 days before the study, lower panels—SPECT/CT of ^188^Re-6D2 mAb at 2, 8, and 24 hrs after injection, respectively; (c) patient from Phase Ia study with large pelvic mass: from left to right—^18^FDG PET/CT 10 days before the study, SPECT/CT of ^188^Re-6D2 mAb 8 hrs after injection.

Whole body planar scintigraphy showed no uptake of ^188^Re-6D2 in the normal melanized tissues like the retina of the eye, skin, and melanized neurons in brain (Figure 1(a)). Tumor targeting was visible on both whole-body planar scintigraphy and SPECT/CT. SPECT/CT of ^188^Re-6D2 demonstrated targeting of various lesions: mediastinal and lung (Figure 1(b)), pelvic (Figure 1(c)), cutaneous, muscular, and nodal metastases. The highest uptake in the tumors was observed at 2 and 8 h after injection, with the tumor still visible 24 h after injection (Figure 1(b)).

### 3.3. Dynamics of ^18^FDG Uptake after Treatment

**Figure 2:**
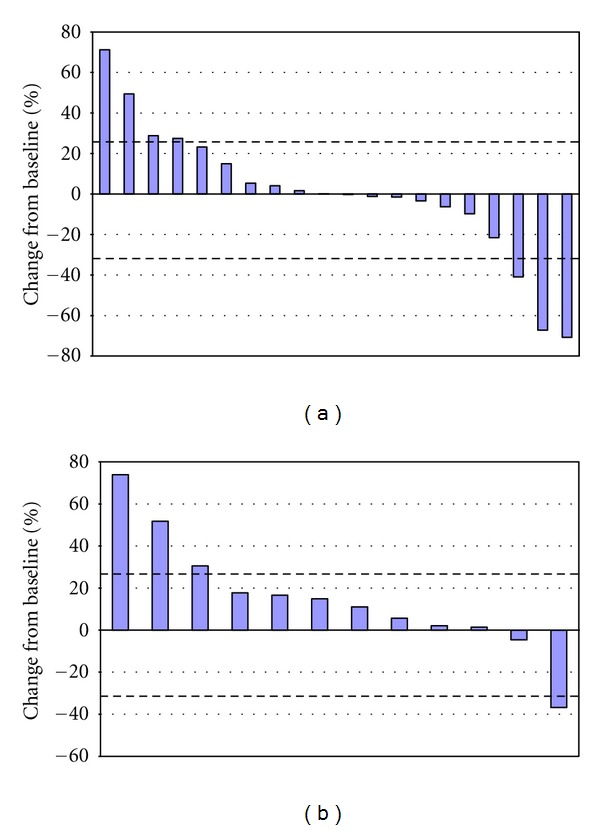
Waterfall plots of ^18^FDG uptake in the tumors after treatment with ^188^Re-6D2: (a) % change from baseline of SUV_max⁡_; (b) % change from baseline of SUV_mean_. Every bar represents an individual patient. Dashed lines at 30% and −30% represent the separation from stable metabolic disease and progressive metabolic disease or partial metabolic response, respectively.

^18^FDG PET/CT scans were performed prior to treatment and between 2 and 6 weeks after treatment for each patient. The maximum and the mean standardized uptake values (SUV_max⁡_ and SUV_mean_, resp.) were summed at baseline and at the last after treatment assessment to determine the percentage change from baseline. The results are shown in Figure 2. SUV_max⁡_ was calculated for all 20 patients, whereas SUV_mean_ was only calculated for 12 patients. The results from these analyses are in concordance with the changes seen in target lesions after treatment per-RECIST. Based upon the SUV_max⁡_ percent change from baseline, 15 patients experienced stable metabolic disease, 2 patients had progressive metabolic disease, and 3 patients experienced a partial metabolic response. Thirteen patients had stable metabolic disease. One patient from phase Ia and one from phase Ib experienced stable metabolic disease or partial metabolic responses in 100% of identified lesions. A complete metabolic response was observed in a single lesion in two additional patients, one from each study.

### 3.4. Pharmacokinetics of ^188^Re-6D2

The effective half-life of ^188^Re-6D2 was 12.2 h and the biological half-life 46.7 h (See Supplementary Table S1 available online at http://dx.doi.org/10.1155/2013/828329). The preload with 10–50 mg of unlabeled 6D2 had no effect on ^188^Re-6D2 effective or biological half-lives compared to ^188^Re-6D2 alone. Serum concentrations of 6D2 were quantifiable in all patients in both studies up to 48 hs after infusion of ^188^Re-6D2.

### 3.5. Dosimetry to Normal Organs

The results from dosimetry calculations for ^188^Re-6D2 are presented in Table 2. The radiation doses of ^188^Re-6D2 (up to 54 mCi) administered in these two studies were determined to be safe when compared to the tolerance of normal tissue to therapeutic irradiation causing 50% complications in 5 years [15]. The kidneys and bone marrow had the highest radiation absorbed doses relative to MTDs for normal organs. At the highest doses administered in phase 1b, the kidneys absorbed 13.2 ± 1.9% of the maximum tolerated dose and the bone marrow absorbed 8.5 ± 0.9%. These are the potential dose limiting organs. Calculation of the total dose which could be delivered to normal organs by 100 mCi ^188^Re-6D2 demonstrated that doses to the critical organs would be 5 times below TD 50/5 and compare favorably to those from ibritumomab tiuxetan and tositumomab (Table 3).

### 3.6. Tumor Response to ^188^Re-6D2 Administration and Survival

Although the efficacy was not the primary objective of these studies, antitumor activity of ^188^Re-6D2 was evaluated within the confines of a phase I study (Supplementary Table S2). The 10 mCi ^188^Re-6D2 in Phase Ia was not considered a therapeutic dose but was sufficient for imaging and pharmacokinetic analysis. After receiving 10 mCi, 9 patients demonstrated stable disease in target lesions at the conclusion of the 6-week followup period. At the conclusion of the 6-week followup period 3 patients met the RECIST criteria of stable disease and 8 patients had progressive disease. This is an encouraging observation as all patients had previously failed at least two standard therapies. During Phase Ib 4 patients met the criteria for stable disease through week 6. During continued followup one patient had an overall response of stable disease through followup week 22. Two patients continued to have an overall response of stable disease through week 14. Supplementary Figure S1 represents the data for the best percent change from baseline for the sum of the longest diameters (SLD) for the target lesions from each patient. The target lesions for 15 patients were considered stable disease by RECIST, 2 patients had partial responses in their target lesions, and 2 patients had progressive disease. The nontarget lesions in 8 patients showed progression and 9 patients developed new lesions (5 patients had both new lesions and nontarget progression).

**Figure 3:**
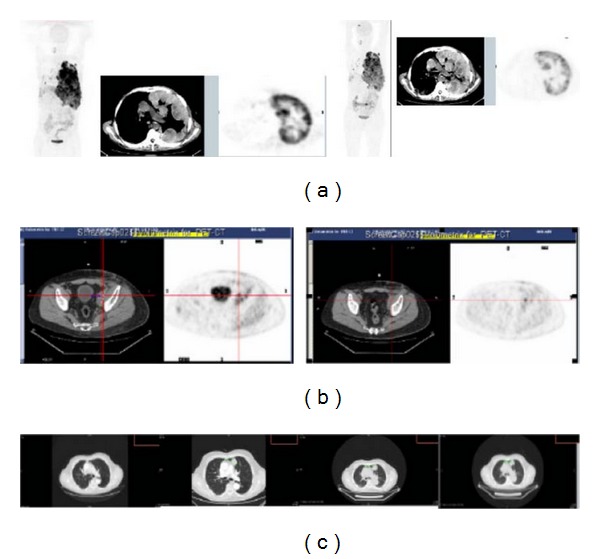
Tumor response to ^188^Re-6D2 mAb in patients. (a) ^ 18^FDG PET/CT of a patient from Phase Ia: left panel—10 days before the study showing coronal and transaxial views, right panel—7 days after the dose of ^188^Re-6D2 mAb; (b) patient from Phase Ib with a stable disease who received 30 mCi/16 mg ^188^Re-6D2 mAb: left panel—^18^FDG PET/CT 10 days before the study; right panel—^18^FDG PET/CT 24 weeks after the study; (c) patient from Phase Ib with progressive disease who received 54 mCi/47 mg ^188^Re-6D2 mAb: panels from left to right—CT 6 months prior to study; 1 month prior to study; at the time of ^188^Re-6D2 mAb administration; 1 month after administration.

Figure 3(a) shows ^18^FDG PET/CT of a patient with massive lung and pleural involvement 10 days before the administration of ^188^Re-6D2 mAb and 7 days after the 10 mCi dose. There was significantly decreased uptake in the tumor 7 days after ^188^Re-6D2 mAb administration corresponding most likely to increased tumor necrosis seen on CT. The tumor necrosis could have been the result of radiation, considering the high uptake of the ^188^Re-6D2 mAb in the lung mets, or a reflection of the natural history of the disease.  Figure 3(b) shows the tumor in a patient who received 30 mCi/16 mg ^188^Re-6D2 mAb and whose tumor was stable at 24 weeks after treatment. At the time of manuscript preparation, this patient was still alive with nonprogressive disease for 17 months.  Figure 3(c) displays one tumor mass in the lung parenchyma of a patient with progressive disease who received 54 mCi/47 mg ^188^Re-6D2 mAb.

A post hoc pooled analysis of overall survival (OS) performed in May 2011 established a median duration of OS at 13 months and mean OS—at 15.6 months. Ten out of 17 (59%) patients had an overall survival greater than 12 months, 3 patients (18%) greater than 24 months with one patient continued to be followed at month 42. Four patients still being followed as of May 2011 were at 22, 23, 25, and 42 weeks after ^188^Re-6D2 mAb administration.

### 3.7. Toxicity, Adverse Effects, and HAMA

During the phase Ib no toxicities were observed for the administered doses including the maximum dose of 54 mCi ^188^Re-6D2 manufactured during the study, suggesting that the doses of ^188^Re-6D2 were well below MTD. All patients in both studies were negative for HAMA at baseline. Supplementary Table S3 displays the dynamics of the HAMA response in patients after receiving ^188^Re-6D2 mAb in both studies. In the phase 1a study, 5 of 13 patients developed a positive HAMA response at 2 weeks after administration with this number decreasing to 3 patients at 6 weeks. Three patients who were HAMA positive at week 2 became negative by week 6. In the second trial, all 7 patients were negative for HAMA at 2 weeks after infusion, with 2 patients developing a positive response at 6 weeks and another patient at 18 weeks. The 2 patients with a positive HAMA response at week 6 did not have any additional follow-up visits to determine if the response returned to negative. The one patient developing a positive HAMA response at week 18 returned to HAMA negative at week 26. There appeared to be no correlation between the ^188^Re-6D2 dose and the prevalence of a HAMA response. Treatment with ^188^Re-6D2 was safe and well tolerated in both studies (See Supplementary Table S4). No significant trends were noted in the types of adverse effects (AEs) at any dose level. In both studies serious AEs were considered to be unrelated to ^188^Re-6D2.

## 4. Discussion

Even in the era of B-RAF inhibition, there continues to be an enormous need for effective “broad spectrum” therapies for MM which would be suitable for all patients diagnosed with the disease. In 1981 DeNardo et al. reported a curative therapy of murine melanoma with ^131^I-labeled mAbs against P-51 murine melanoma [16]. RIT of melanoma moved into the clinical trial in 1985 when 50% tumor reduction was observed in a patient treated with ^131^I-labeled Fab' fragments of a mAb against high molecular weight melanoma-associated antigen [17]. Despite early successes during the 80s, RIT of melanoma did not develop into a clinical modality for a variety of reasons that included disappointing results in clinical trials of different solid tumors during that time.

The availability of novel mAbs and radionuclides with optimal emission characteristics encouraged us to revisit the RIT for MM. We targeted melanin which is a novel antigen for radioimmunotherapy with an IgM mAb 6D2. IgMs are often overlooked in RIT despite fast blood clearance, ability to trigger ADCC and CDC immune responses, and early encouraging data in patients [18]. Though the doses of melanin-binding mAb ^188^Re-6D2 were limited by the activity of the ^188^W/^188^Re generators at the time of the study, the combined survival data for the two studies demonstrated that the median survival of patients receiving ^188^Re-6D2 was approximately 13 months which is longer than the 8.5 months for the MM patients receiving standard care. Although this result must be interpreted cautiously, the finding is encouraging. Importantly, no uptake in the healthy melanized tissues was observed which confirmed our prior observations in the melanoma animal models [10]. The treatment was not accompanied by the severe toxic effects. On the contrary, toxicity was very mild with no hematological AEs observed even in patients receiving the highest doses of ^188^Re-6D2. Several factors contribute to the nontoxic nature of ^188^Re-6D2: (1) fast clearance of ^188^Re-6D2 from the blood which prevents harmful irradiation of bone marrow; (2) relatively short physical half-life of ^188^Re (17.0 hrs) in comparison with 2.8 and 8 days for ^90^Y and ^131^I used in ibritumomab tiuxetan and tositumomab, respectively; (3) ^188^Re nonresidualizing nature which results in its fast excretion through the kidneys; (4) the absence of 6D2 cross-reactivity with normal tissues leading to very low uptake of 6D2 in nontarget tissues. In this regard, the dosimetry calculations showed that up to 100 mCi ^188^Re-6D2 could be safely administered to the patients which should further improve the therapeutic results.

HAMA in the majority of patients was transient and not dependent on the unlabeled mAb dose. In this regard it is important to emphasize that the mAb used in ibritumomab tiuxetan and tositumomab for treatment of NHL is also murine. One can suggest that the radiolabeled murine mAb to melanin can be administered safely to melanoma patients for up to two doses. The work on the conversion of the murine mAb into the human-mouse chimera which would allow for multiple administrations of the radiolabeled mAb to MM patients is currently ongoing in our laboratories.

Our study has some limitations with major ones being relatively small number of patients which precludes statistical analyses of the data and not achieving the maximum tolerated dose for the ^188^Re-6D2 due to the technical restrictions of the ^188^W/^188^Re generators available to us at the time of the trial. The high activity/high specific activity ^188^W/^188^Re generators have become available commercially after the completion of this study which will ensure that higher doses could be administered to patients in the follow-up trials.

In conclusion, Phase I trials of ^188^Re-6D2 mAb to melanin in patients with MM demonstrated tumor targeting and safety of the drug, as well as prolongation in survival. These results are encouraging and suggest the need for further investigation of this reagent by itself or in combination with other therapies.

## Supplementary Material

Supplementary Figure S1 shows the waterfall plot of the best percentage change from baseline of the sum of the longest diameters (SLD) for the target lesions.
Supplementary Table S1 shows the effective and biological half-lives of ^188^Re-6D2 mAb in MM patients.
Supplementary Table S2 shows the RECIST overall response and lesion response at week 6 post-treatment visit.
Supplementary Table S3 shows the HAMA response from phase 1a and phase 1b studies.
Supplementary Table S4 shows the summary of adverse effects (AE).

## Figures and Tables

**Table 1:** Patients demographics.

	Phase Ia	Phase Ib
Age, mean (min, max)	64.2 (45, 83)	64.7 (54, 80)
Male	10 (76.9%)	5 (71.4%)
Caucasian	13 (100%)	7 (100%)
Stage IV	13 (100%)	6 (85.7%)
Prior therapies		
Chemotherapy	11 (84.6%)	3 (42.9%)
Immunotherapy	9 (69.2%)	6 (85.7%)
Radiotherapy	3 (23.1%)	4 (57.1%)
Surgery	13 (100%)	7 (100%)

**Table 2:** Radiation-absorbed doses to organs from ^188^Re-6D2 (mSv/MBq).

Cohort	Liver	Spleen	Kidneys	Bone marrow	Urine bladder wall	Whole body
Phase 1a						
Cohort 1	0.88 ± 0.13	0.86 ± 0.09	1.35 ± 0.05	0.09 ± 0.01	0.88 ± 0.23	0.16 ± 0.01
Cohort 2	0.89 ± 0.20	0.67 ± 0.05	1.11 ± 0.22	0.11 ± 0.02	1.01 ± 0.12	0.15 ± 0.03
Cohort 3	0.90 ± 0.08	0.73 ± 0.14	1.59 ± 0.01	0.11 ± 0.0	0.86 ± 0.14	0.13 ± 0.02
Cohort 4	1.04 ± 0.30	0.67 ± 0.30	1.29 ± 0.12	0.14 ± 0.03	1.02 ± 0.08	0.16 ± 0.04
Phase 1b						
Cohort 1	0.92 ± 0.20	0.93± 0.32	1.99 ± 0.17	0.11± 0.01	0.61 ± —*	0.14 ± 0.01
Cohort 2	0.77 ± 0.25	1.02 ± 0.35	1.75 ± 0.25	0.10 ± 0.01	0.95 ± 0.47	0.15 ± 0.03
Tositumomab	0.82	1.14	1.96	0.65	0.64	0.24
Ibritumomab tiuxetan	4.8	9.4	0.1	1.3	0.9	0.5

*Only one patient had sufficient data for calculation of urine bladder wall dose.

**Table 3:** Radiation-absorbed doses of ^188^Re-6D2 compared to tositumomab, ibritumomab tiuxetan, and normal organ tolerance TD 5/5 (cGy).

	Liver	Kidneys	Bone marrow	Urine bladder wall
Tositumomab 81 mCi	246	588	195	192
Ibritumomab tiuxetan 30 mCi	533	11	144	100
^ 188^Re-6D2 40–60 mCi	130	305	17	158
^ 188^Re-6D2 Estimated 100 mCi	344	650	42	350
Normal Organ Tolerance	3000	2300	200	6500
