# Supplementary material for: Safety and Efficacy of 188-Rhenium-Labeled Antibody to Melanin in Patients with Metastatic Melanoma
Source: J Skin Cancer. 2013 Jan 10;2013:828329. doi: 10.1155/2013/828329 (PMC3556872; doi:10.1155/2013/828329)
Supplement: Supplementary file 1 — Supplementary Figure S1 shows the waterfall plot of the best percentage change from baseline of the sum of the longest diameters (SLD) for the target lesions. Supplementary Table S1 shows the effective and biological half-lives of 188Re-6D2 mAb in MM patients. Supplementary Table S2 shows the RECIST overall response and lesion response at week 6 post-treatment visit. Supplementary Table S3 shows the HAMA response from phase 1a and phase 1b studies. Supplementary Table S4 shows the summary of adverse effects (AE). [file 828329.f1.pdf]

Supplementary data for MS “Safety and efficacy of 188-Rhenium-labeled antibody to melanin in patients with metastatic melanoma’ by M. Klein et al.

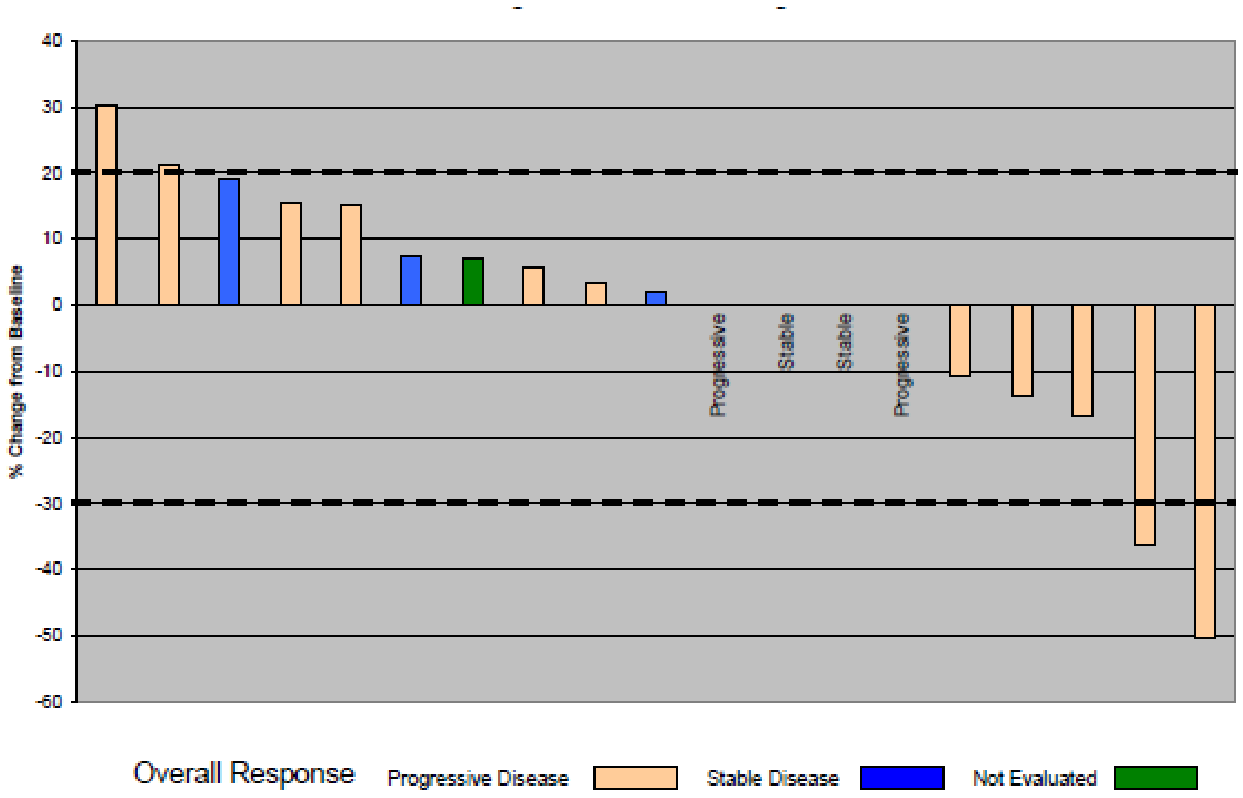

Fig. S1 Waterfall plot of the best percentage change from baseline of the sum of the longest diameters (SLD) for the target lesions. The dashed line at 20% depicts the separation of stable and progressive disease and the dashed line at -30% depicts the separation between stable disease and partial response. Every bar represents an individual patient. The patient’s overall tumor response is represented by the color of the bar. Patients that have a zero percent change do not have an associated bar. One patient was excluded from the plot due to missing post-treatment measurements.

Table S1 Effective and biological half-lives of ^{188}Re-6D2 mAb in MM patients

Cohort	T_{eff}, hours, mean	T_{eff}, hours, range	T_{bio}, hours, mean	T_{bio}, hours, range
Phase 1a Cohort 1	12.2	10.6 - 13.4	46.7	28.2 - 63.3
Phase a Cohort 2	12.1	11.8 - 12.4	42.5	38.6 - 45.8
Phase 1a Cohort 3	12.5	11.7 - 14.1	52.8	37.5 - 82.6
Phase 1a Cohort 4	12.3	11.8 - 12.8	45.4	38.6 - 51.8
Phase 1b Cohort 1	12.8	11.9 - 13.8	53.0	39.7 - 72.0
Phase 1b Cohort 2	12.0	11.3 -12.6	41.7	33.5 - 48.6

Table S2 RECIST Overall response and lesion response at week 6 post-treatment visit

	Phase 1b	Phase 1a
Overall Response	n=5	n=13
Complete response	0 (0%)	0 (0%)
Partial response	0 (0%)	0 (0%)
Stable disease	3 (60.0%)	3 (23.1%)
Progressive disease	2 (40.0%)	8 (61.5%)
Not evaluated	0 (0 %)	2 (15.4%)
Target lesion response		
Complete response	0 (0%)	0 (0%)
Partial response	0 (0%)	1 (7.7%)
Stable disease	4 (80.0%)	9 (69.2%)
Progressive disease	0 (0%)	2 (15.4%)
Not evaluated	1 (20.0%)	1 (7.7%)
Non-target lesion response		
Complete response	0 (0%)	1 (7.7%)
Partial response	0 (0%)	0 (0%)
Stable disease	2 (40.0%)	1 (7.7%)
Progressive disease	2 (40.0%)	5 (38.5%)
Not evaluated	0 (0%)	2 (15.4%)
NA	1 (20.0%)	4 (30.8%)
Appearance of new lesions		
No new lesions since baseline	3 (60.0%)	8 (61.5%)
Appearance of 1 or more new lesions	2 (40.0%)	5 (38.5%)
Not evaluated	0 (0%)	0 (0%)

Table S3 HAMA response from phase 1a and phase 1b studies

	Phase 1b	Phase 1a
Screening	n=7	n=13
Positive	0 (0%)	0 (0%)
Negative	7 (100.0%)	13 (100.0%)
Week 2		
Positive	0 (0%)	5 (41.7%)
Negative	7 (100.0%)	7 (58.3%)
Week 6		
Positive	2 (28.6%)	3 (25.0%)
Negative	4 (57.1%)	9 (75.0%)
Week 18		
Positive	1 (14.3%)	NA
Negative	1 (14.3%)	NA
Week 22		
Positive	1 (14.3%)	NA
Negative	1 (14.3%)	NA

After week 6, only weeks where positive HAMA results were obtained are displayed

Table S4 Summary of adverse effects (AE)

Number of Patients (%)	Phase Ia	Phase Ib
Any AE	7 (53.8%)	6 (85.7%)
Any Grade 3 AE	3 (23.1%)	1 (14.3%)
Any Grade 4 AE	0 (0%)	0 (0%)
Any SAE	3 (23.1%)	2 (28.6%)
Any DLT	0 (0%)	0 (0%)
AEs Leading to Treatment Discontinuation	0 (0%)	0 (0%)
AEs Leading to Death*	1 (11.1%)	1 (14.3%)

*Not related to the study
